# Effect of Proanthocyanidins from Grape Seed Extract on Benign Prostatic Hyperplasia

**DOI:** 10.3390/nu17010073

**Published:** 2024-12-28

**Authors:** Fortuna Iannuzzo, Elisabetta Schiano, Maria Maisto, Anna Schettino, Noemi Marigliano, Anella Saviano, Adel Abo Mansour, Asif Jilani Iqbal, Francesco Maione, Gian Carlo Tenore, Ettore Novellino

**Affiliations:** 1 Department of Pharmacy, University of Chieti-Pescara G. D’Annunzio, 66100 Chieti, Italy; fortuna.iannuzzo@unich.it; 2 Inventia Biotech—Healthcare Food Research Center s.r.l., Strada Statale Sannitica KM 20.700, 81020 Caserta, Italy; elisabettaschiano@inventiabiotech.com; 3 Department of Pharmacy, University of Naples Federico II, Via Domenico Montesano 49, 80131 Naples, Italy; maria.maisto@unina.it; 4 ImmunoPharmaLab, Department of Pharmacy, School of Medicine and Surgery, University of Naples Federico II, Via Domenico Montesano 49, 80131 Naples, Italy; anna.schettino2@unina.it (A.S.); noemi.marigliano@outlook.com (N.M.); anella.saviano@unina.it (A.S.); a.j.iqbal@bham.ac.uk (A.J.I.); 5 Department of Clinical Laboratory Sciences, College of Applied Medical Sciences, King Khalid University, Abha 62521, Saudi Arabia; aabomansour@kku.edu.sa; 6 Department of Cardiovascular Sciences, College of Medicine and Health, University of Birmingham, Birmingham B15 2TT, UK; 7 Faculty of Medicine and Surgery, Catholic University of the Sacred Heart, 00168 Rome, Italy; ettore.novellino@unicatt.it

**Keywords:** benign prostatic hyperplasia, grape seeds, proanthocyanidins, *Vitis vinifera*

## Abstract

Background/Objectives: Benign prostatic hyperplasia (BPH) is one of the most common chronic diseases affecting the urinary tract that occurs mainly in men over 40 years of age. Among the natural therapies, proanthocyanidins (PACs), which can treat a wide range of immune-mediated inflammatory diseases (IMIDs), have been shown to play an important role in the treatment of pathologies concerning prostate health. In this regard, the present study aimed to evaluate the different bioactivities of a grape seed extract (GSE), rich in polymeric PACs, and its version processed under alkaline conditions (ATGSE), characterized by a higher content of oligomeric PACs, in an animal model of BPH induced by subcutaneous injection of testosterone (1 mg/mouse). Methods: These latter were divided into a control group (vehicle, olive oil), a BPH group (testosterone 1 mg/mouse), and four treatment groups treated with GSE (500 mg/kg) and ATGSE (125, 250, 500 mg/kg) by oral gavage. At the experimental endpoint (4 weeks), hematological and biochemical analyses of blood and tissues were performed. Results: Data showed that oral administration of ATGSE (250 mg/kg) was significantly more effective than GSE in reducing prostate (*p* ≤ 0.0001) and seminal vesicle (*p* ≤ 0.0001) weight. Moreover, ATGSE exhibited enhanced effectiveness in significantly reducing PSA levels (*p* ≤ 0.0001 vs. GSE) and the expression of key pro-inflammatory cyto-chemokines in prostate and seminal vesicles homogenates. Conclusions: These findings pave the way for the clinical application of ATGSE as a nutraceutical and/or functional food.

## 1. Introduction

Benign prostatic hyperplasia (BPH) is a frequent chronic disease of the urinary tract that mainly occurs in men over 40 years and whose incidence increases progressively with age [1]. It is marked by increased proliferation of smooth muscle cells, stromal cells, and epithelial cells, resulting in an enlarged prostate. This can lead to obstruction of the urethra and the development of lower urinary tract symptoms (LUTS), such as urinary urgency, increased urinary frequency, nocturia, dysuria, weak urinary stream, and incomplete bladder emptying [2,3]. Although the cause of BPH remains unclear, several lines of evidence suggest that prostate size in older men is influenced by circulating androgen hormones, including testosterone and dihydrotestosterone (DHT) [4,5]. DHT is synthesized in the prostate from circulating testosterone through the action of the enzyme 5-α-reductase [6] and represents a more potent androgen compared to testosterone because it binds with a higher degree of affinity to the androgen receptor, which regulates the expression of genes that promote the growth and survival of stromal and epithelial cells in the prostate [7]. However, the excessive proliferation of these cells by androgens, especially DHT, is considered to be an important cause of BPH [8]. In this context, a research study carried out by Pejic et al., [9] showed that testosterone and DHT accumulate in the prostatic stroma in patients with BPH, and there exists a direct relationship between the concentration of testosterone and DHT in prostate tissue and prostate size. Inflammation is another key process implicated in the development of prostate diseases, which is a protective mechanism against tissue damage or pathogens. In this context, previous research has demonstrated that the degree of localized inflammation in the prostate of patients with BPH is related to increased prostate size [5]. This process causes increased synthesis and secretion of inflammatory cytokines and macrophages in response to inflammatory damage, as well as an increase in mediators that play a crucial role in the biosynthesis of arachidonic acid (e.g., thromboxane, prostaglandins, and leukotrienes) and oxidative stress [10,11]. The most used test for the diagnosis of BPH is the measurement of prostate-specific antigen (PSA) levels. The serum PSA level helps distinguish between normal and pathological conditions [12]. Elevated serum PSA levels have been frequently detected in patients with male reproductive system pathologies, such as prostate cancer, BPH, and inflammatory prostate disease [13]. The treatment of BPH is mainly focused on pharmacological therapies and surgical intervention. Surgery is more reliable, but it can lead to many complications, such as urinary incontinence, erectile dysfunction, bleeding, infection and urethral stricture. Pharmacological treatment can be classified into α1-adrenergic receptor antagonists (doxazosin, terazosin, and tamsulosin) and 5-α-reductase inhibitors (finasteride and dutasteride). However, the use of these medications can lead to serious side effects, such as erectile dysfunction, decreased sexual desire and reduction of seminal volume in the ejaculate (for 5-α reductase inhibitors), orthostatic hypotension, asthenia, dizziness, impotence, and retrograde ejaculation (for α-1-adrenergic receptor antagonists) [14]. Therefore, research interest has increasingly focused on the development of alternative therapeutic strategies, with improved safety and efficacy, based on the use of natural remedies and/or nutraceutical and functional foods for the prevention and adjuvant treatment of BPH. In this regard, several studies have reported the beneficial effects of proanthocyanidins (PACs) in the treatment and prevention of diseases affecting prostate health [14,15,16]. This evidence is in line with the potential to treat a broad range of immune-mediated inflammatory diseases (IMIDs). PACs are polymers formed by the condensation of monomer units of flavan-3-ols and are widely known for their antioxidant, anticancer, anti-inflammatory, and antimicrobial activities [17]. One of the major sources of PACs are grape seeds, which are waste products of great importance in the alimentary (but also nutraceutical and food supplement) industry because they are the most widely cultivated worldwide and represent a source that is still rich in bioactive compounds [18,19,20]. In this regard, a study conducted by Lei et al. [21] on rats with testosterone-induced BPH demonstrated that treatment with PACs derived from grape seeds effectively reduced prostate size, improved elevated androgen levels, and regulated the expression of pro-inflammatory cytokines. However, the bioactivity and bioavailability of PACs can be variable depending on their degree of polymerization. Monomeric and oligomeric PACs are more bioavailable than those characterized by a higher degree of polymerization. This property leads to increased bioactivity, as low molecular weight PACs can reach the target tissues more effectively and exert more pronounced biological effects [22,23,24]. Based on this consideration, in our previous study, we demonstrated that a food-grade treatment enables the depolymerization of PAC polymers from grape seed extract (GSE) (*Vitis vinifera* L.) into lower molecular weight oligomers and monomers by alkaline treatment [25]. This treatment led to the development of an innovative nutraceutical product named ATGSE (alkaline treatment of GSE), enriched in oligomeric PACs. Therefore, the main objective of the present study was to evaluate the potential beneficial effects of ATGSE, investigating whether its oligomeric PAC content, due to their greater bioavailability and bioactivity, could promote more significant therapeutic benefits in reducing prostate enlargement and modulating the inflammatory responses associated with BPH compared to the polymeric molecules present in GSE. For this purpose, GSE and ATGSE were administered daily by oral gavage to mice with BPH induced by subcutaneous injection of testosterone. At the experimental endpoint (4 weeks), blood samples were collected by an intracardiac puncture to evaluate PSA values, while prostate, seminal vesicles, and testicles were micro surgically removed from mice and weighed. Finally, a proteome profiler mouse cytokine array kit was used on prostate and seminal vesicle homogenates to assess the modulation of the main pro-inflammatory cyto-chemokines.

## 2. Materials and Methods

### 2.1. Materials

Testosterone propionate (c.n.: T1875) and olive oil (c.n.: 75348) were purchased from Sigma-Aldrich Co. (now under Merck, Darmstadt, Germany)) and the Mouse PSA ELISA Kit (c.n.: E-EL-M0961) was purchased from Elabscience (Milan, Italy). The proteome profiler mouse cytokine array kit (c.n.: ARY006) was obtained from R&D System (Milan, Italy). GSE from *Vitis vinifera* L. was purchased from MB-Med S.r.l. (Turin, Italy). Unless otherwise stated, all the other reagents were from BioCell (Milan, Italy).

### 2.2. Sample Preparation and Dosage

The study was designed to evaluate the beneficial effects of GSE and ATGSE in mice with induced BPH. The ATGSE sample was prepared following the procedure described in a previous study [25]. In the first phase of the research, the therapeutic effect of the two matrices was evaluated at the highest dosage of 500 mg/kg to determine their relative efficacy. Based on this preliminary analysis, a dose assessment was conducted on the sample that demonstrated greater efficacy, testing concentrations of 250 and 125 mg/kg. The dosages were selected with the aim of employing an intermediate dose (250 mg/kg) that could be translated into a safe and easily administrable daily dose for humans. Additionally, lower and higher doses (125 and 500 mg/kg) were also tested to evaluate a potential dose-dependent response and to explore the efficacy of treatment across a wide range of concentrations.

### 2.3. Cell Culture and Viability

Murine macrophage J774A.1 cell line was grown in Petri culture dishes (100 × 20 mm) using Dulbecco’s modified Eagle’s medium (DMEM) supplemented with 10% Fetal Bovine Serum (FBS), 2 mM L-glutamine, 100 U/mL penicillin [26], 100 μg/mL streptomycin [27], 25 mM 4-(2-hydroxyethyl)-1-piperazineethanesulfonic acid (HEPES) [28], and 130 μg/mL sodium pyruvate [29] in a humidified atmosphere containing 5% carbon dioxide at 37 °C. Cell viability [30] was assessed as previously described [31], employing a colorimetric 3-(4,5-dimethylthiazol-2-yl)-2,5-diphenyltetrazolium bromide (MTT) assay. In brief, J774A.1 cells (5 × 10^3^ per well) were plated in 96-well plates and incubated overnight. Cells were then treated with GSE and ATGSE (0.0001–1 mg/mL). After 4 and 24 h, 10 µL of MTT solution (5 mg/mL in phosphate-buffered saline, PBS; pH 7.4) was added to each well and the plates were incubated for 3 h at 37 °C. Then, the medium was removed, and the obtained formazan [32] crystals were solubilized in 150 µL of DMSO for 15 min. The spectrophotometric absorbance was recorded at 540 nm using a microtiter enzyme-linked immunosorbent assay reader (Multiskan^TM^GO Microplate Spectrophotometer; Thermo Scientific^TM^, Waltham, MA, USA). The percentage of cell viability was calculated using the following formula: OD of treated cells/OD of control × 100.

### 2.4. Animals

All animal care and experimental protocols complied with national and European community guidelines, international and national law and policies and were approved (Authorization number: 354/2019-PR and 1/2024-PR) by the Institutional Animal Care and Use Committee (IACUC) (EU Directive 2010/63/EU for animal experiments, and the Basel declaration including the 3Rs concept). Male CD-1 mice (10–14 weeks of age, 25–30 g of weight) were purchased from Charles River (Milan, Italy). Animals were maintained in specific pathogen-free conditions, housed in ventilated cages with controlled temperature and humidity, on a 12 h light/dark cycle and provided *ad libitum* access to standard laboratory chow diet and sterile water. Experimental groups were randomized, and evaluations were conducted by researchers blinded to the treatment groups. All procedures were made to minimize the number of animals used (n = 6 per group) and reduce their suffering during the experiments.

### 2.5. In Vivo Model and Drug Administration

After acclimatization, animals were randomly divided into six experimental groups (six animals per group): (i) Control group (Ctrl), (ii) BPH, (iii) BPH + GSE 500 mg/kg, (iv) BPH + ATGSE 125 mg/kg, (v) BPH + ATGSE 250 mg/kg, and (vi) BPH + ATGSE 500 mg/kg group (Table 1). Mice in BPH experimental groups received subcutaneous injections of testosterone propionate (1 mg/mouse) dissolved in olive oil for 28 days [33,34,35], while the control group received olive oil alone. GSE and ATGSE were dissolved in distilled water (vehicle) and administered daily by oral gavage (p.o., 300 μL/mouse) for 28 days. Body weights were taken the day before the treatment (baseline) and monitored weekly throughout the experimental period (Supplementary Figure S1). At the experimental endpoint (4 weeks), mice were anesthetized using 5% isoflurane (inhalation) to enable blood sampling via intracardiac puncture. Animals were subsequently sacrificed through exposure to a mixture of CO_2_ (70%) and O_2_ (30%). Prostates, testicles, and seminal vesicles were carefully excised by microsurgery, cleared of connective tissue, and weighed [36]. Whole blood samples were left to stand for 30 min at room temperature and then centrifugated at 10,000 rpm for 5 min. The resulting sera were transferred into a 1.5 mL tube and stored at −80 °C for up to 30 days until ex vivo analysis.

### 2.6. Elisa Assay: Detection of PSA Levels in Serum

PSA levels were evaluated to determine the degree of hyperplasia caused by testosterone injection in the prostate. For this purpose, serum PSA levels were quantified using a commercially available Elisa kit, following a previously described protocol [37]. The PSA Elisa kit (c.n.: E-EL-M0961, Elabscience) is designed for the quantitative measurement of total PSA levels. In brief, 100 μL of serum, diluted standards, quality controls, and dilution buffer (blank) were added to a pre-coated plate containing monoclonal anti-PSA for 2 h. After washing, 100 μL of biotin-labelled antibody was added, and the plate was incubated for an additional hour. After washing, 100 μL of streptavidin—horseradish peroxidase (HRP) conjugate was added to the plate and incubated for 30 min in the dark. Subsequently, 100 μL of substrate solution followed by stop solution was added as the final step. Absorbance was measured at 450 nm using a microplate reader (Multiskan^™^ GO Microplate Spectrophotometer; Thermo Scientific^™^). Antigen concentrations in the samples were determined using a PSA standard curve, normalized to serum levels, and expressed as ng/mL.

### 2.7. Cytokines and Chemokines Protein Array

At the experimental endpoint (4 weeks), mice from all experimental conditions were sacrificed and prostates and seminal vesicles were promptly removed and collected into a 2 mL tube for preservation in liquid nitrogen, followed by storage at −80 °C. The collected tissues were homogenized in ice-cold tris(hydroxymethyl)aminomethane hydrochloride (Tris-HCL) buffer (20 mM, pH 7.4) containing 1 mM ethylenediaminetetraacetic acid (EDTA), 1 mM ethylene glycol-bis(β-aminoethyl ether)-N,N,N′, N′-tetra acetic acid (EGTA), 1 mM phenylmethylsulfonyl fluoride (PMSF), 1 mM sodium orthovanadate, and one tablet of Complete^TM^ Protease Inhibitor Cocktail (Roche, c.n.: 04693132001) per 50 mL of buffer. According to the manufacturer’s guidelines, equal volumes (1.5 mL) of pooled prostate or seminal vesicle homogenates from each experimental group were incubated with the pre-coated proteome profiler array membranes. Dot plots were visualized using an enhanced chemiluminescence detection kit and Image Quant 400 GE Healthcare software (GE Healthcare, Milan, Italy), and the resulting data were quantified using GS 800 imaging densitometer software (Biorad, Segrate, Italy) [31].

### 2.8. Statistical Analysis

Statistical analyses were conducted in accordance with international guidelines for experimental design and data analysis in pharmacology [38] and for data sharing and presentation in preclinical pharmacology [39,40]. Results are expressed as means ± S.D. Normality was assessed before performing one- or two-way ANOVA, followed by Bonferroni’s or Dunnett’s post hoc tests for multiple comparisons, with significance set at *p* ≤ 0.05. Data analysis was performed using GraphPad Prism 8.0.2 software (San Diego, CA, USA). The sample size was determined to achieve an alpha level of 0.05 and a power of 0.8. Randomization and group allocation were based on animal weight to minimize unwanted variability through data normalization. No animals and associated ex vivo samples were excluded from the analysis. In vivo study was designed to generate groups of equal size (n = 6 of independent values), using randomization and blinded assessments.

## 3. Results

To assess the potential cytotoxicity, ATGSE and GSE samples were tested on the J774A.1 murine macrophage cell line. The evaluation of the potential beneficial effects of GSE and ATGSE for BPH treatment was carried out in an in vivo model of BPH induced by subcutaneous injection of testosterone (1 mg/mouse). The experimental groups were divided into a control group (vehicle, olive oil), a BPH group (testosterone 1 mg/mouse), and four treatment groups administered with GSE (500 mg/kg) and ATGSE (125, 250, 500 mg/kg) by oral gavage for four weeks. At the experimental endpoint, blood samples were obtained by intracardiac puncture to evaluate PSA levels by enzyme-linked immunosorbent assay (ELISA) analysis, and prostates, testicles, and seminal vesicles were weighted after being isolated from mice by microsurgery. Finally, an Elisa Spot was performed on prostate and seminal vesicle homogenates to assess the modulation of the multiple pro-inflammatory cyto-chemokines.

### 3.1. Safety Profile of GSE and ATGSE on Murine Macrophages Cell Line J774A.1

**Figure 1:**
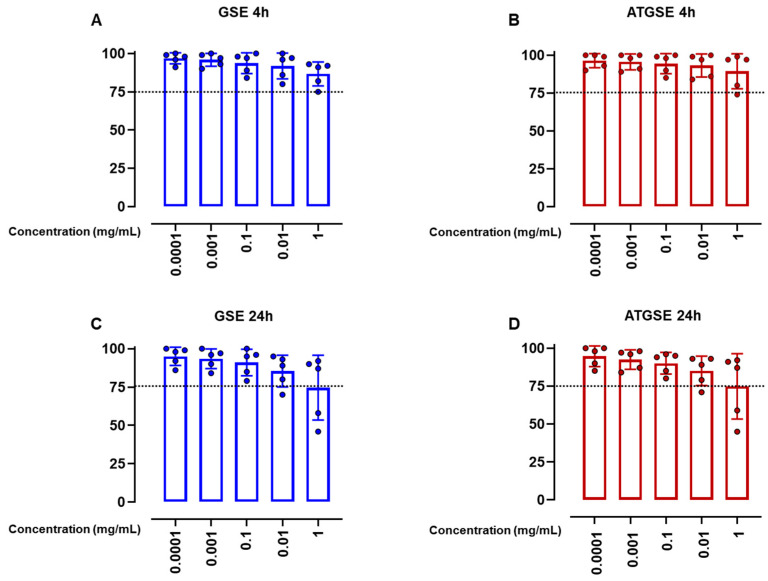
An in vitro cytotoxic assessment was carried out using MTT assay on murine macrophage J774A.1 cell line after 4 h (**A**, **B**) and 24 h (**C**,**D**) of treatment with the selected concentrations of GSE and ATGSE (0.0001–1 mg/mL). The dotted lines represent 75% cell viability. Results are expressed as cell viability (% of control) and are shown as means ±  S.D. from five independent experiments.

In vitro cytotoxicity evaluation (conducted in compliance with ISO 10993-5:2009 (Biological Evaluation of Medical Devices—Part 5: Tests for In Vitro Cytotoxicity) revealed a safe profile for GSE and ATGSE on murine macrophage J774A.1 cell line. Results, represented in Figure 1, show that both GSE and ATGSE did not have any cytotoxic effect independent of the several concentrations tested (Figure 1A–D).

### 3.2. Assessment of Prostate Weight and PSA Levels

**Figure 2:**
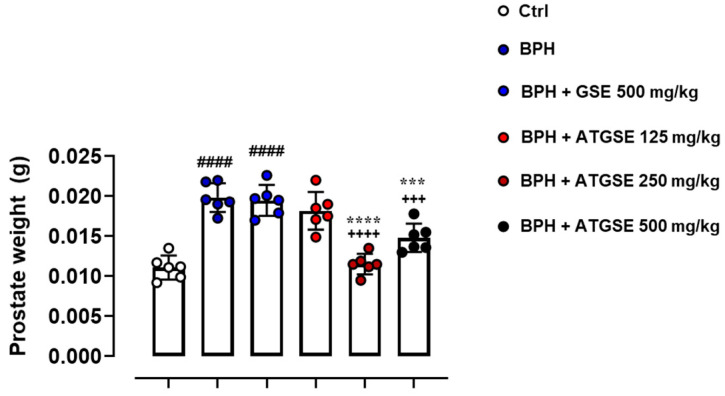
Evaluation of prostate weight at the experimental endpoint (4 weeks). Data are expressed as grams (g) and presented as means ± S.D. (n = 6 for each experimental group). Statistical analysis was performed using one-way ANOVA followed by Bonferroni’s post hoc test for multiple comparisons: ^####^ *p* ≤ 0.0001 vs. Ctrl; ^+++^ *p* ≤ 0.001, ^++++^ *p* ≤ 0.0001 vs. BPH; *** *p* ≤ 0.001, **** *p* ≤ 0.0001 vs. BPH + GSE 500 mg/kg.

Four weeks following the treatment, prostate weight was significantly increased in the testosterone group (BPH) compared to the control group (vehicle) (Figure 2). Furthermore, the testosterone and GSE-treated group at a dose of 500 mg/kg (BPH + GSE 500 mg/kg) showed a significant increase compared to the control group (*p* ≤ 0.0001), but no meaningful difference was observed compared to the BHP group. In the three experimental groups administered with testosterone concomitantly with ATGSE (BPH + ATGSE 125 mg/kg, 250 mg/kg, and 500 mg/kg), a significant reduction in prostate weight was observed at doses of 250 mg/kg and 500 mg/kg compared to the BPH group (*p* ≤ 0.0001 for 250 mg/kg, and *p* ≤ 0.001 for 500 mg/kg) and compared to the BPH + GSE 500 mg/kg group (*p* ≤ 0.0001 for 250 mg/kg and *p* ≤ 0.001 for 500 mg/kg). Moreover, prostate weight, normalized to mice body weight (prostate weight/body weight), exhibited the same trend as prostate weight expressed in grams. These data and representative images of prostate tissues, collected from all experimental conditions, at the experimental endpoint (4 weeks) are provided in Supplementary Figure S2A,B. Collectively, our results indicate that ATGSE at a dose of 250 mg/kg showed the highest reduction in prostate weight.

**Figure 3:**
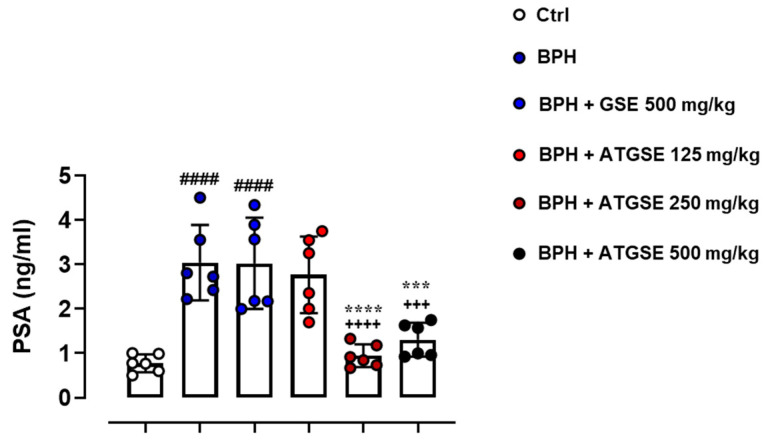
Measurement of PSA levels at the experimental endpoint (4 weeks). Data are expressed as ng/mL and presented as means ± S.D. (n = 6 for each experimental group). Statistical analysis was performed using one-way ANOVA followed by Bonferroni’s post hoc test for multiple comparisons: ^####^ *p* ≤ 0.0001 vs. Ctrl; ^+++^ *p* ≤ 0.001, ^++++^ *p* ≤ 0.0001 vs. BPH; *** *p* ≤ 0.001, **** *p* ≤ 0.0001 vs. BPH + GSE 500 mg/kg.

PSA levels were assessed in serum samples using a commercial ELISA kit (Figure 3). The standard PSA value in the control group (vehicle) was 0.772 ± 0.201 ng/mL. In the BPH experimental group, a significant increase in PSA of 3.037 ± 0.849 ng/mL was observed compared to control (*p* ≤ 0.0001). In the same way, the BPH + GSE 500 mg/kg group showed a significant increase in PSA levels (3.023 ± 1.025 ng/mL) in comparison to the control group (*p* ≤ 0.0001), but no significant difference was observed compared to BPH group. In contrast, the BPH + ATGSE experimental groups (250 and 500 mg/kg) revealed a significant reduction in PSA levels (2.766 ± 0.860 ng/mL and 0.945 ± 0.258 ng/mL, respectively), compared to both the BPH (*p* ≤ 0.0001 for 250 mg/kg and *p* ≤ 0.001 for 500 mg/kg) and BPH + GSE 500 mg/kg experimental groups (*p* ≤ 0.0001 for 250 mg/kg and *p* ≤ 0.001 for 500 mg/kg). The BPH + ATGSE 125 mg/kg group showed a PSA value of 2.766 ± 0.860 ng/mL, but no significant variation was found compared to the other experimental groups. Consistent with the previous findings, treatment with ATGSE at the dose of 250 mg/kg was the most effective in reducing PSA levels.

### 3.3. Evaluation of Seminal Vesicle and Testicle Weight

**Figure 4:**
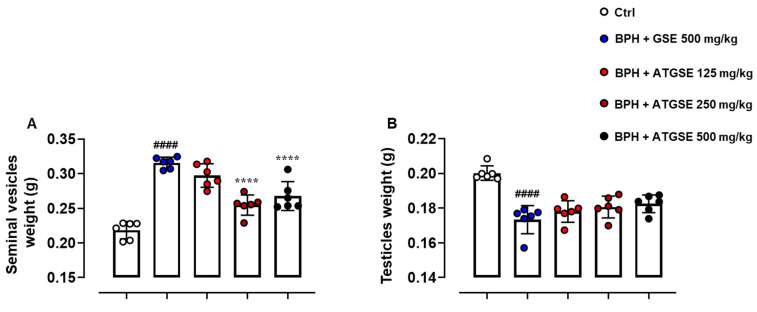
Evaluation of seminal vesicle (**A**) and testicle (**B**) weight at the experimental endpoint (4 weeks). Data are expressed as grams (g) and presented as means ± S.D. (n = 6 for each experimental group). Statistical analysis was performed using one-way ANOVA followed by Bonferroni’s post hoc for multiple comparisons: ^####^ *p* ≤ 0.0001 vs. Ctrl; **** *p* ≤ 0.0001 vs. BPH + GSE 500 mg/kg.

The examination of seminal vesicles and testicle weight was performed for the following experimental groups: control, BPH and GSE 500 mg/kg, BPH and ATGSE, at three different doses (125, 250, 500 mg/kg). Since previous experiments on prostate weight and PSA levels revealed no significant differences between the BPH experimental group and the BPH + GSE 500 mg/kg group, BPH was not included in this analysis. The results obtained are shown in Figure 4. In the BPH + GSE 500 mg/kg group, the weight of the seminal vesicles significantly increased after 28 days of treatment in comparison to the control group (*p* ≤ 0.0001). Furthermore, in the three experimental groups administered with testosterone concomitantly with ATGSE (125 mg/kg, 250 mg/kg, and 500 mg/kg), a significant decrease in seminal vesicle weight was observed at the dose of 250 and 500 mg/kg compared to the BPH + GSE 500 mg/kg group (*p* ≤ 0.0001). Conversely, the analysis of testicle weight showed a significant reduction in the BPH + GSE 500 mg/kg group (*p* ≤ 0.0001), however, none of the three doses of ATGSE were effective in reversing this pathological condition. Representative images of seminal vesicles and testicles, collected from all experimental conditions, at the experimental endpoint (4 weeks) are shown in Supplementary Figure S3A,B.

### 3.4. Unveiling the Immunological Mediator Profile on Prostate and Seminal Vesicle Homogenates: Focus on Pro- and Anti-Inflammatory Cyto-Chemokine Profile

**Figure 5:**
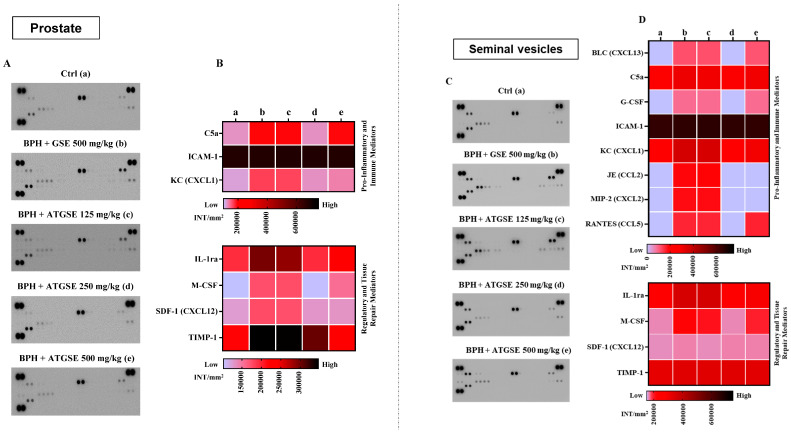
Densitometric analysis is presented as a heatmap with dots highlighting the most significantly modulated cyto-chemokine mediators in prostate (**A**,**B**) and seminal vesicle homogenates (**C**,**D**). Data (expressed as INT/mm^2^) are presented as median ± S.D. (double-gradient) of positive spots from two independent experiments, each conducted with n = 6 mice for pooled experimental group: Ctrl (**a**), BPH + GSE 500 mg/kg (**b**), BPH + ATGSE 125 mg/kg (**c**), BPH + ATGSE 250 mg/kg (**d**), BPH + ATGSE 500 mg/kg (**e**). Elisa Spot assay statistical analysis (reported in the text) was performed by using two-way ANOVA followed by Dunnett’s post hoc for multiple comparisons.

Inflammation is one of the key drivers of BPH disease pathogenesis. We sought to measure local inflammatory mediators produced in response to the initiation of disease. All detectable pro-inflammatory cyto-chemokines from prostate and seminal vesicle homogenates for the following experimental groups: control, BPH and GSE 500 mg/kg, BPH and ATGSE at three different doses (125, 250, 500 mg/kg) (Figure 5A,C) were assayed using a proteome profiler cytokine array kit. The quantification of pro-inflammatory cyto-chemokines in prostate and seminal vesicle homogenates for each experimental condition is presented as a heatmap (Figure 5B,D, respectively) and expressed as integrated intensity (INT)/mm^2^ (membranes associated with the densitometric analysis are reported Figure 5A,C). Furthermore, the inflammatory mediators detected by the Elisa Spot assay were divided into two groups: pro-inflammatory and immune mediators, and regulatory and tissue repair mediators. Consistent with previous results, the administration of ATGSE selectively mitigated the pro-inflammatory onset in both homogenates analyzed. In particular, the analysis of the cyto-chemokine profile on prostate homogenates showed that the BPH + GSE 500 mg/kg group compared to the control group (vehicle) significantly enhanced the expression of all cyto-chemokines except ICAM-1. Furthermore, the testosterone group and ATGSE-treated group at doses of 250 and 500 mg/kg compared to the BPH + GSE 500 mg/kg group induced a notable reduction of the following mediators: IL-1ra (*p* ≤ 0.0001), KC (*p* ≤ 0.0001 for 250 mg/kg and *p* ≤ 0.001 for 500 mg/kg), M-SCF (*p* ≤ 0.0001 for 250 mg/kg and *p* ≤ 0.01 for 500 mg/kg), SDF-1 (*p* ≤ 0.0001), and TIMP-1 (*p* ≤ 0.0001). In the case of C5a, a potent neutrophil chemoattractant, a striking reduction was observed only at a dose of 250 mg/kg (*p* ≤ 0.0001) and in this case, additionally, there was no significant modulation of ICAM-1. Furthermore, the analysis of the cyto-chemokines performed on seminal vesicle homogenates revealed the expression of the same inflammatory mediators detected on prostate homogenates, in addition to other mediators such as (BLC, G-CSF, JE, MIP-2, and RANTES). The testosterone group and ATGSE-treated group at the doses of 250 and 500 mg/kg, compared to BPH + GSE 500 mg/kg group resulted in a significant reduction of the following mediators: IL-1ra (*p* ≤ 0.0001), KC(*p* ≤ 0.0001), JE (*p* ≤ 0.0001), and MIP-2 (*p* ≤ 0.0001) and M-SFC (*p* ≤ 0.0001 for 250 mg/kg and *p* ≤ 0.05 for 500 mg/kg), whereas a prominent reduction of BLC (*p* ≤ 0.0001), C5a (*p* ≤ 0.0001), G-CSF (*p* ≤ 0.0001), and RANTES (*p* ≤ 0.0001) mediators was observed only at the dose of 250 mg/kg. In this analysis, consistent with previous studies, there was no considerable modulation of ICAM-1, along with SDF-1 and TIMP-1 (with *p* ≤ 0.01 vs. Ctrl). Collectively, these results confirmed that ATGSE 250 mg/kg was the most effective dose modulating the expression of several pro-inflammatory mediators.

## 4. Discussion

BPH is a pathological condition characterized by abnormal proliferation of epithelial and stromal cells in the prostate gland. It occurs in men over 40 years of age and is one of the common causes of LUTS [41]. As the prostate enlarges, it constricts the urethra, leading to various symptoms such as weak urinary stream, incomplete bladder emptying, nocturia, dysuria, and bladder outlet obstruction [42]. Although the molecular mechanisms associated with the pathogenesis of this metabolic disorder have not yet been fully elucidated [43], several factors such as inflammatory mediators, hormonal, dietary factors, and oxidative stress have been implicated in its etiology [44]. Currently, pharmacological therapies conventionally used for the treatment of BPH include α-1-adrenergic receptor antagonists (doxazosin, terazosin, and tamsulosin), which relieve LUTS by relaxing the smooth muscle in the prostate and bladder, and 5-α-reductase inhibitors (dutasteride and finasteride), which inhibit the conversion of testosterone to DHT in the prostate [15,45]. Although these conventional drugs have proven effective in the treatment of BPH, the adverse side effects associated with their use (impotence, gynecomastia, orthostatic hypotension, abnormal ejaculation), have led to increased research of alternative solutions for managing this disease. Although surgery represents an additional option, it can lead to various complications and high costs. In this regard, research has increasingly focused on developing safe and effective alternative therapeutic strategies based on the use of natural remedies for the prevention and treatment of BPH [15,16,46]. In particular, much evidence of the role of PACs from grape seeds in the treatment of diseases affecting prostate health has been reported [21,47,48]. Raina et al. investigated the chemo-preventive efficacy of GSE in prostate cancer using a transgenic mouse model (TRAMP). The results showed that oral administration of GSE led to a 46% reduction in urogenital organ weight in comparison to the control group. In addition, GSE reduced the cell proliferation index by 50% and increased the number of apoptotic cells eightfold, while the expression of cyclins (A, B1, and E) and kinases (Cdk2, Cdk6, and Cdc2), proteins that regulate cell growth, decreased by 80% [47]. Others have evaluated the protective potential of GSE against carrageenan-induced abacterial prostatitis in rats. They indicated that the administration of the extract significantly reduced pro-inflammatory cytokines such as TNF-α, IFN-γ, and IL-6, as well as the prostatic index and prostatic acid phosphatase activity, biomarkers associated with hyperplasia and prostate tissue damage [48]. The only study available in the literature that specifically focused on the beneficial effects of GSE in the BPH treatment was conducted by Lei et al., using a mouse model of testosterone-induced BPH in castrated rats. The authors demonstrated that GSE led to a significant reduction in DHT levels and a decrease in 5-α-reductase activity. Compared to finasteride, used as a positive control, GSE demonstrated greater efficacy in reducing pro-inflammatory cytokines (IL-1β, IL-6, TNF-α, and COX-2) and enhancing the activity of antioxidant enzymes (SOD and GPX) [21]. Other studies have demonstrated that the molecular size and degree of polymerization of PACs have a major influence on their bioavailability and bioactivity, which are usually higher for low molecular weight compounds [49]. Monomeric and oligomeric molecules have a simpler chemical structure and a smaller molecular size, allowing them to penetrate cell membranes more easily, which improves their absorption and distribution to target tissues where they can exert their biological activities. These molecules can more readily interact with cellular receptors, rapidly activating cellular signaling pathways and achieving faster and more significant biological effects [50,51]. In a previous study, we demonstrated that a food-grade alkaline treatment promotes the depolymerization of PAC polymers from GSE (*Vitis vinifera* L.) into lower molecular weight oligomers and monomers by alkaline treatment. This treatment led to the development of an innovative nutraceutical product, named ATGSE, enriched in potentially more bioavailable and bioactive monomeric and oligomeric PACs [25]. At present, there are limited studies assessing the impact of PACs from grape seeds in the treatment of BPH, and no evidence has been provided to compare the differential biological efficacy of their monomers and polymers in managing this condition. Here, we aimed to evaluate whether the higher fraction of monomeric PACs in the ATGSE formulation could exert a more significant effect on the reduction of pathological conditions associated with BPH than the polymeric fraction contained in GSE. Therefore, GSE and ATGSE were administered to mice in an in vivo model of BPH induced by a testosterone subcutaneous injection. At the experimental endpoint, blood samples were collected by intracardiac puncture to evaluate PSA levels, while prostate, seminal vesicles, and testicles were micro surgically removed from mice and weighed. The results demonstrated that oral administration of ATGSE at the dose of 250 mg/kg resulted in a significant reduction in prostate weight compared to BPH-treated animals. Since treatment with GSE at a dose of 500 mg/kg did not show any significant difference compared to the BPH group alone, no relevant reduction in prostate weight was observed. The reduction in prostate volume obtained after ATGSE administration could be attributed to the effect of oligomeric PACs, which act by lowering DHT levels and inhibiting the activity of 5-α-reductase, as reported in various studies [48,52]. A review conducted by Kumar et al. (2022) also emphasized that monomeric PACs from tea are highly effective in preventing prostate cancer and reducing prostate volume. Studies in TRAMP mice (transgenic adenocarcinoma of the prostate in mice) have shown that the administration of monomeric PACs resulted in a significant reduction in prostate weight. This effect is attributed to the capacity of PAC monomers to inhibit cell proliferation and induce apoptosis, thereby slowing tumor growth and reducing prostate volume. Although BPH and prostate cancer have different pathological conditions, they share common mechanisms, such as inflammation and uncontrolled cell proliferation, both contributing to increased prostate volume. Therefore, the ATGSE nutraceutical formulation could have a similar effect and reduce prostate volume even in non-oncological contexts. In contrast, GSE administered at a higher dose was less effective in achieving these therapeutic outcomes, since polymeric PACs may be ineffective in regulating prostate growth mechanisms compared to monomers. The results obtained for prostate weight are consistent with the PSA evaluation. PSA, which is exclusively synthesized by prostate cells, is currently considered a sensitive and specific marker for prostate diseases, including BPH, prostatitis, and cancer [53]. Serum levels are typically elevated in patients affected by these pathologies [13,54]. Therefore, the results presented here showed that testosterone injection leads to a significant increase in PSA levels (*p* < 0.001 vs. Ctrl), while oral administration of ATGSE (250 mg/kg) significantly reduced PSA to levels nearly comparable to the control group. In contrast, the BPH + GSE 500 mg/kg group showed no significant differences compared to the BPH group. This result confirms the positive effect of the monomeric PACs. The significant reduction in prostate weight observed after ATGSE treatment correlates closely with the decrease in PSA levels, indicating the efficacy of the treatment in reducing prostate hyperplasia and restoring physiological prostate volume. It is well known that the prostate, along with testicles and seminal vesicles, supports the healthy functioning of the male reproductive system. These organs play a crucial role in the production of seminal fluid, so their dysfunction can increase the risk of infertility, which is an important consequence of BPH [55]. Treatments for BPH, such as 5-alpha reductase inhibitors (e.g., finasteride), work by reducing DHT levels, indirectly influenced by testicular hormone production. These treatments can decrease prostate size and alleviate BPH symptoms, demonstrating the importance of the testicles in the hormonal cascade contributing to BPH. Therefore, analysis of testicle weight showed a significant reduction in the BPH + GSE 500 mg/kg group compared to the control group. As reported in the literature, testosterone subcutaneous injection in mouse models leads to a reduction in testicle volume, so the significant decrease in testicular weight observed in the BPH + GSE 500 mg/kg group can be ascribed exclusively to the effects of testosterone. This is further supported by the absence of significant differences in prostate weight and PSA levels between this group and the BPH group [34]. Furthermore, none of the three ATGSE doses were able to reverse this pathological condition. This outcome could be due to the limited ability of ATGSE bioactive compounds to penetrate the testicular microenvironment and exert their effects, or to the possibility that the administered doses were inadequate to counteract the physiological changes induced by BPH. Although the seminal vesicles themselves are not directly implicated in BPH, their proximity and shared ductal pathways mean that BPH symptoms (like urinary retention and sexual dysfunction) may indirectly involve the seminal vesicles. Conversely, analysis of seminal vesicle weight showed a significant reduction in BPH + ATGSE 250 mg/kg, compared to the group treated with testosterone and GSE at the dose of 500 mg/kg. These results further highlight the greater protective effect of the ATGSE formulation in countering the pathological processes associated with BPH. Another important factor involved in the progression of BPH is inflammation, an immunological defense mechanism against pathogens or tissue damage [5]. Several studies have shown that there is a significant association between prostate size and acute or chronic inflammation [56]. Furthermore, the anti-inflammatory role of PACs in the treatment of inflammatory conditions is widely reported in the literature [57]. Therefore, to analyze the anti-inflammatory effects of GSE and ATGSE on BPH, the expression of the main pro-inflammatory cyto-chemokines, which are generally upregulated during disease onset and progression, were monitored [58,59,60]. Testosterone injected in the BPH experimental group induced an inflammatory response in both prostate and seminal vesicle homogenates, leading to an increase in the expression of various inflammatory mediators. Specifically, in the prostate homogenates, testosterone injection resulted in a significant upregulation of cyto-chemokines such as C5a, ICAM-1, M-SCF, SDF-1, IL-1α, KC, and TIMP-1. Furthermore, in the seminal vesicle homogenates, a prominent expression of additional inflammatory mediators, including BLC, G-CSF, IL-1ra, JE, MIP-2, and RANTES, was detected. This suggests that although both tissues are exposed to the same androgenic stimulation, they respond differently due to the presence of distinct cell populations, such as lymphocytes, neutrophils, and macrophages, thereby modulating the production of different cytokines and chemokines [61]. However, the oral administration of ATGSE at the dose of 250 mg/kg resulted in a marked reduction of the expression of pro-inflammatory mediators such as C5a, IL-1ra, KC, M-SCF, SDF-1, and TIMP-1 in prostate homogenates, and BLC, C5a, G-CSF, IL-1ra, KC, M-SCF, MIP-2, and RANTES in seminal vesicle homogenates, compared to the GSE group. This finding clearly highlights the ability of ATGSE to modulate inflammatory mediator levels in the local microenvironment, thereby reducing localized inflammation associated with BPH and leading to an overall reduction of prostate gland volume and hyperreactivity.

## 5. Conclusions

In conclusion, ATGSE, an efficient nutraceutical formulation enriched in oligomeric PACs, may represent a promising and effective therapeutic and/or adjuvant agent for BPH treatment. In an in vivo model of BPH, this nutraceutical formulation showed a significant reduction in prostate and seminal vesicle weights, as well as in PSA levels. Furthermore, ATGSE also revealed a protective effect against the inflammatory process triggered during the development of the pathology, by reducing the expression of pro-inflammatory cyto-chemokines in the involved anatomical districts. However, further studies will be conducted to evaluate its efficacy and safety in humans, unravelling its clinical rational use as nutraceutical and/or functional food.

## Figures and Tables

**Table 1:** Schematic representation of in vivo experimental groups and drug administration.

Exp. Group	Group Name	Testosterone	GSE	ATGSE
I	Control	-	-	-
II	BPH	1 mg/mouse	-	-
III	BPH + GSE	1 mg/mouse	500 mg/kg	-
IV	BPH + ATGSE	1 mg/mouse	-	125 mg/kg
V	BPH + ATGSE	1 mg/mouse	-	250 mg/kg
VI	BPH + ATGSE	1 mg/mouse	-	500 mg/kg

## Data Availability

The data used to support the findings of this study are included in the article.

## Supplementary Materials

The following supporting information can be downloaded at: https://www.mdpi.com/article/10.3390/nu17010073/s1, Figure S1: Body weight monitoring of animals over a 4-week period in a testosterone-induced benign prostatic hyperplasia model. Weight measurements (expressed as g) were recorded weekly to assess potential changes associated with the hypertrophy induction and any systemic effects of testosterone administration. Figure S2: Variation in prostate weight relative to body weight (**A**) at the experimental endpoint (4 weeks) in the testosterone-induced benign prostatic hyperplasia model. Representative images of the prostate at the experimental endpoint, illustrating morphological differences beetween groups (**B**). Data are expressed as grams (g) and presented as means ± S.D. (n = 6 for each experimental group). Statistical analysis was performed using one-way ANOVA followed by Bonferroni’s post-hoc test for multiple comparisons: ^####^ *p* ≤ 0.0001 vs. Ctrl; ** *p* ≤ 0.01, **** *p* ≤ 0.0001 vs. BPH + GSE 500 mg/kg. Figure S3: Representative images of seminal vesicles (**A**) and testicles (**B**) collected at the experimental endpoint (4 weeks) in the testosterone-induced benign prostatic hyperplasia model, highlighting the effects of testosterone injection and ATGESE (125 mg/kg, 250 mg/kg and 500 mg/kg) treatment on their morphology.
