# Supplementary material for: Effect of Proanthocyanidins from Grape Seed Extract on Benign Prostatic Hyperplasia
Source: Nutrients. 2024 Dec 28;17(1):73. doi: 10.3390/nu17010073 (PMC11723264; doi:10.3390/nu17010073)
Supplement: Supplementary file 1 [file nutrients-17-00073-s001.zip › nutrients-3363057-supplementary.pdf]

Supplementary Materials

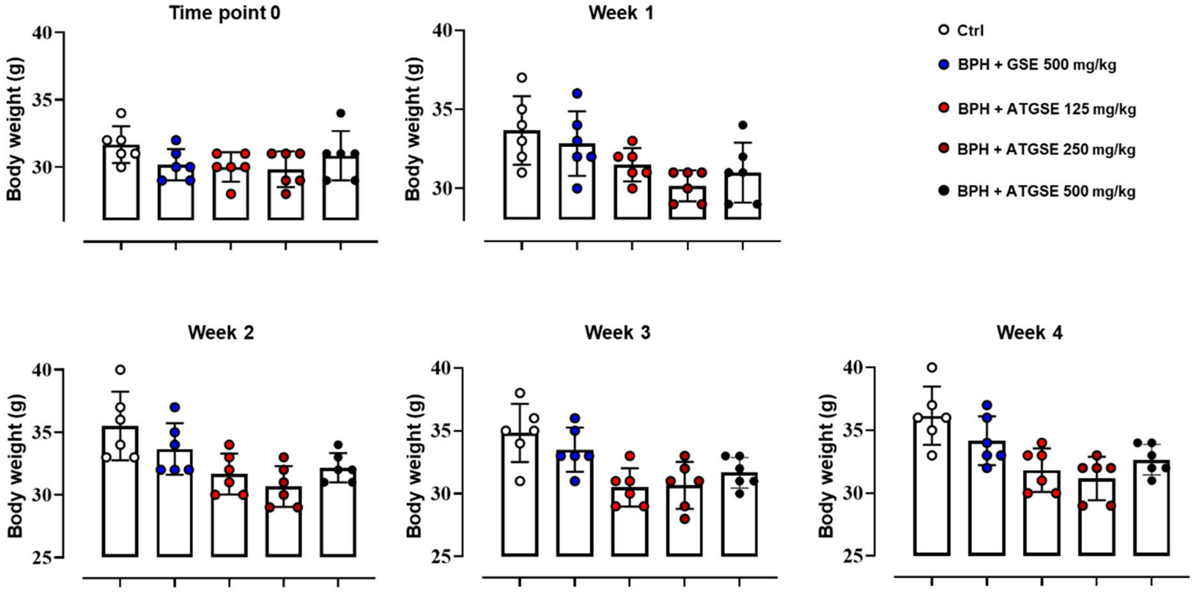

Supplementary Figure S1. Body weight monitoring of animals over a 4-week period in a testosterone-induced benign prostatic hyperplasia model. Weight measurements (expressed as g) were recorded weekly to assess potential changes associated with the hypertrophy induction and any systemic effects of testosterone administration.

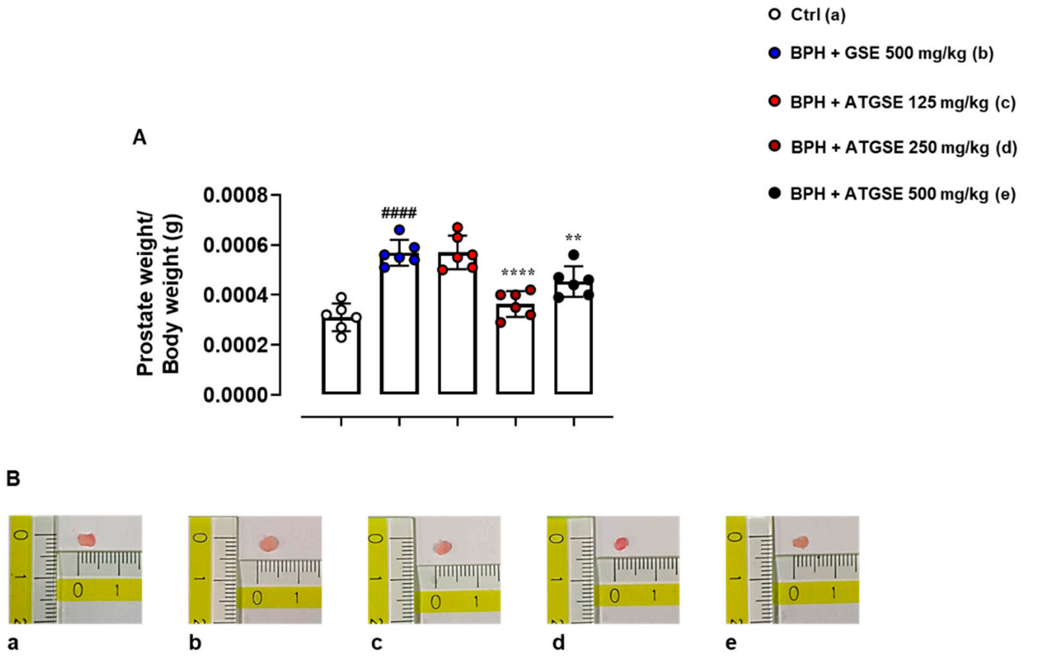

Supplementary Figure S2. Variation in prostate weight relative to body weight (**A**) at the experimental endpoint (4 weeks) in the testosterone-induced benign prostatic hyperplasia model. Representative images of the prostate at the experimental endpoint, illustrating morphological differences beetween groups (**B**). Data are expressed as grams (g) and presented as means ± S.D. (n=6 for each experimental group). Statistical analysis was performed using one-way ANOVA followed by Bonferroni's post-hoc test for multiple comparisons: ####$p \leq 0.0001$ *vs* Ctrl; **$p \leq 0.01$, ****$p \leq 0.0001$ *vs* BPH + GSE 500 mg/kg.

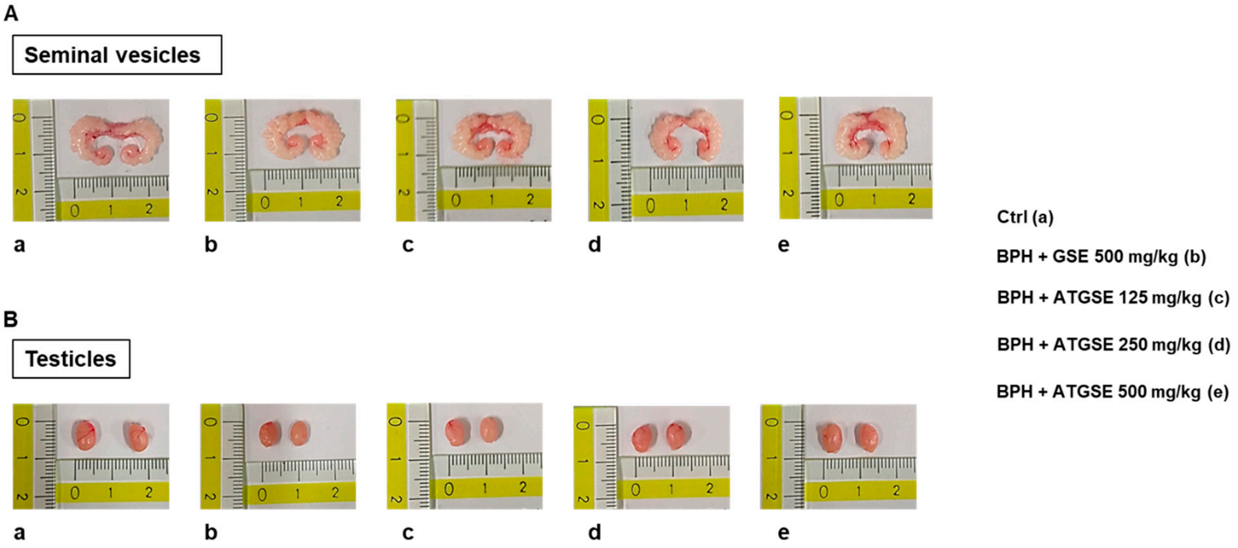

Supplementary Figure S3. Representative images of seminal vesicles (**A**) and testicles (**B**) collected at the experimental endpoint (4 weeks) in the testosterone-induced benign prostatic hyperplasia model, highlighting the effects of testosterone injection and ATGESE (125 mg/kg, 250 mg/kg and 500 mg/kg) treatment on their morphology.
